# The Study of Clinical Profile of Patients With Mucormycosis During COVID-19 Pandemic in Tertiary Care Hospital

**DOI:** 10.7759/cureus.47065

**Published:** 2023-10-15

**Authors:** Santosh Kumar, Anita, Anand Dev, Abhay Kumar, Santosh Kumar Nayan, Siddharth Singh

**Affiliations:** 1 Emergency Medicine, Indira Gandhi Institute of Medical Sciences, Patna, IND; 2 Microbiology, Indira Gandhi Institute of Medical Sciences, Patna, IND

**Keywords:** rhino-orbital-cerebral mucormycosis, diabetes mellitus, steroid, mucormycosis, covid-19

## Abstract

Introduction: Mucormycosis has gained a huge number of cases in the second wave of post COVID-19 infection, which may be attributed to increased awareness, advancement in diagnostic techniques, and an increase in the prevalence of predisposing factors. This study evaluated the pattern, risk factors, and clinical profile of patients with mucormycosis during the second wave of the COVID-19 pandemic.

Methods: A prospective observational study was conducted in the Department of Emergency Medicine of a dedicated tertiary care hospital for COVID-19. The Institutional Ethics Committee approved the study. One hundred five patients diagnosed with mucormycosis were included from June 2021 to December 2021. Informed consent was obtained from the patients. Data on demography, clinical features, predisposing factors, co-morbid conditions, and microbiological samples were obtained and analyzed.

Results: Out of 105 patients, 71 were male, and 34 were female. The patient presented with mucormycosis between the 2nd and 3rd week of post COVID-19 infection. Incidence was mainly seen in patients in their fifties, mostly associated with diabetes mellitus (DM) (53.30%), oxygen administration (80%), and previous use of steroids (45.71%). Predominantly, males were more affected. The most common presentation was headache (50.47%), orbital pain with restricted ocular movement (47.67%), proptosis (42.85%), and diminished vision (41.90%). Rhino-orbital-cerebral mucormycosis (ROCM) was the most common presentation of mucormycosis, while only five cases of pulmonary mucormycosis were found.

Conclusion: ROCM was the most common presentation of mucormycosis between the second and third week of post COVID-19 infection. Diabetes mellitus and inadvertent use of steroids were major predisposing factors. Therefore, a high degree of suspicion and early diagnosis with initiation of treatment is warranted in cases of mucormycosis in post COVID-19 infection.

## Introduction

COVID-19 is caused by SARS-CoV-2. It can affect any age, but patients with diabetes, steroid use, cancer, cardiovascular disease, and chronic liver disease are at higher risk. The presentation in patients may vary from mild to severe disease, primarily related to respiratory symptoms [1]. In the second wave of COVID-19, there was a deluge of patients with mucormycosis.

Mucormycosis is a fungal infection caused by mucorales fungi such as rhizopus, rhizomucor, mucor, cunninghamella, and lichtheimia. Manifestation of mucormycosis depends on its entry and association with other comorbid conditions. Mucormycosis affects the nasal mucosa, paranasal sinus, orbit, central nervous system, lungs, gastrointestinal system, kidney, and skin. Mucormycosis leads to angioinvasion of tissue, causing arterial thrombosis and tissue destruction [2].

Phagocytes are the primary defense against mucormycosis. Diabetes and steroid therapy are the main risk factors for mucormycosis. Higher glucose levels due to any cause like diabetes, new-onset hyperglycemia, and steroid-induced hyperglycemia lead to an ideal environment of low oxygen levels in tissue, decreasing the phagocytic activity of white blood cells (WBC) and causing immunosuppression. Mortality in mucormycosis ranges from 50% to 80% [2]. The cases of mucormycosis in India during the second wave of the COVID-19 pandemic were 70 times higher than in the remaining world [3]. The mucormycosis burden in India was 0.14 cases per 1,000 population [4]. In a pre-COVID era study, Indian patients were assessed retrospectively for zygomycosis and reported 178 cases, with rhino-orbital-cerebral mucormycosis (ROCM) as the most common infection [5].

Due to its aggressive nature, rapid diagnosis and treatment are essential in mucormycosis cases [6]. Those with uncontrolled diabetes and other co-morbidities are primarily predisposed to ROCM [7]. Several studies also suggested that pulmonary mucormycosis had lower mortality when compared with disseminated infection [8]. Our study aims to evaluate the pattern, risk factors, and clinical profile of patients with mucormycosis during the second wave of the COVID-19 pandemic.

## Materials and methods

This was a prospective observational study conducted from June 2021 to December 2021 at a dedicated tertiary care center for COVID-19. The tertiary care center had 300 dedicated beds for patients with COVID-19 and was well-equipped with intensive care and high-dependency units. This study adhered to the tenets of Helsinki and was carried out after obtaining ethical clearance from the Institutional Ethical Committee (186/IEC/IGIMS/2021). Patients with confirmed cases of mucormycosis were included in the study. Patients who did not give consent to participate were excluded. The admission and treatment were done by the treating physicians as per institute policy.

Data of the patients, including demographics, clinical presentation, comorbid conditions, history of previous drug use, mode of oxygen administration during previous hospitalization, and previous and present documentation of reverse transcription-polymerase chain reaction (RT-PCR) for COVID-19. The details were collected using the standard Case Record Form (CRF). The CRF also had details of the patients about the level of education, occupation, and exposure to the damp area and animal husbandry. Risk factors included organ transplant, AIDS, carcinoma, and IV drug abuse were specifically asked for.

The patient underwent complete general clinical, otorhinolaryngological, ophthalmological examination, and radiological imaging. The following biochemical parameters were extracted: glycosylated hemoglobin, fasting blood sugar, post-prandial blood sugar, complete blood count, inflammatory markers (serum ferritin, C-reactive protein, and erythrocyte sedimentation rate), and renal function test. MRI brain or contrast CT scan of the head, eye, and paranasal sinuses was done to see involvement and severity of infection. The use of contrast was based on the renal status of the patient. The following findings were noted on radiological examination: involvement of paranasal sinuses, involvement of intraocular muscles, intraorbital fat, orbital apex, uptake of contrast, and intracranial extension. The patients were planned for transnasal endoscopic orbital decompression or open surgical debridement depending upon the orbital involvement as evident on radiological and clinical examination. Every patient underwent nasal wash. Samples of nasal wash were sent for microbiological examination. A biopsy was taken from the appropriate disease's site and samples were sent to the Department of Microbiology and Pathology. KOH mount preparation and culture were done in SDA (Sabourd Dextrose Agar media) for fungus and in blood agar medium. Salt pepper-like Growth appeared on SDA media and growth was confirmed by lactose phenol cotton blue (LPCB) mount preparation. A hyaline with broad aseptate hyphae suggested a sample positive for the mucor group of fungi on KOH mount.

All analyses were performed using the SPSS version 20 (IBM Corp., Armonk, NY). Categorical variables were reported as counts and percentages. Continuous data were given as mean ± SD and range or median.

## Results

The male-female ratio across different age categories is as follows: <20 years (male: 0, female: 0), 21-30 years (4:1), 31-40 years (3:1), 41-50 years (22:13), 51-60 years (18:7), 61-70 years (3:2), 71-80 years (3:1), and 81-90 years (male: 0, female: 1). The overall cohort consisted of 105 patients, with 71 (67%) male and 34 (33%) female (Table 1).

**Table 1:** Age and genderwise distribution of patient and elaboration of risk factors categorized by gender

		Male	Female
Age group	<20	00 (0.00%)	00 (0%)
	21-30	04 (5.63%)	01 (2.94%)
	31-40	15 (21.12%)	05 (14.70%)
	41-50	22 (30.98%)	13 (38.23%0
	51-60	18 (25.35%)	07 (20.58%)
	61-70	09 (12.67%)	06 (17.64%)
	71-80	03 (4.22%)	01 (2.94%)
	81-90	00 (0.00%)	01 (2.94%)
	Total (N=105)	71 (67%)	34 (33%)
Risk factors	Diabetes	36 (50.7%)	20 (58.82%)
	Steroid use	34 (47.8%)	14 (41.17%)
	Organ transplant	01 (1.4%)	0
	Carcinoma	0	0
	Neutropenia	0	0
	Iron overload	0	0
	IV drug abuser	0	0
	HIV	0	0

Among the male group (n=71), diabetes was identified as a prevalent risk factor in 36 (50.7%) patients, followed by steroid use in 34 (47.8%) patients. The incidence of organ transplant history was observed in one (1.4%) patient (Table 1).

Among the female group (n=34), diabetes constituted a significant risk factor for 20 (58.82%) patients, and steroid use in 14 (41.17%) patients. At the same time, no patients with carcinoma, neutropenia, iron overload, IV drug abuse, or HIV infection were recorded in either gender. The cumulative risk factor analysis for the entire cohort of 105 patients indicated that diabetes was prevalent in 56 (53.33%) cases, steroid use in 48 (45.71%) cases, and organ transplant history in one (0.95%) case.

Within the male group (n=71), 28 (39.43%) patients were educated below matriculation, 21 (29.57%) patients had attained graduation, 12 (16.90%) patients had completed post-graduation, and 10 (14.08%) patients held professional degree. Within the female group (n=34), 21 (61.76%) had educational qualification below matriculation, seven (20.58%) had achieved graduation, four (11.76%) had acquired post-graduation degree, and two (5.88%) held professional qualification. In the overall cohort of 105 patients, 49 (46.66%) individuals had education level below matriculation, 28 (26.66%) had graduated, 16 (15.23%) had completed post-graduation, and 12 (11.42%) held professional degree (Table 2).

**Table 2:** Literacy profile, duration between COVID-19, and mucormycosis and modality of oxygen use during hospitalization

		Male (71)	Female (34)	Total (n=105)
Education	Below Matriculation	28 (39.43%)	21 (61.76%)	49 (46.66%)
	Graduation	21 (29.57%)	07 (20.58%)	28 (26.66%)
	Post Graduation	12 (16.90%)	04 (11.76%)	16 (15.23%)
	Professional	10 (14.08%)	02 (5.88%)	12 (11.42%)
Duration between COVID-19 and mucormycosis	<10 days	16 (22.53%)	06 (17.64%)	22 (20.95%)
	10-20 days	44 (61.97%)	18 (52.94%)	62 (59.04%)
	>20 days	11 (15.49%)	10 (29.41%)	21 (20.00%)
Oxygen	No Oxygen Support	15 (21.12%)	6 (17.64%)	21 (20.00%)
	Oxygen Mask/Nasal Canula	40 (56.33%)	20 (58.82%)	60 (57.14%)
	BIPAP support	12 (16.90%)	05 (14.70%)	17 (16.19%)
	Ventilator support	05 (7.04%)	02 (5.88%)	7 (6.66%)

Among the male patients (n=71), 16 (22.53%) had a gap between COVID-19 and mucormycosis was less than 10 days, 44 (61.97%) patients had a gap of 10 to 20 days, and 11 (15.49%) had a gap of 20 days of COVID-19 and mucormycosis (Table 2).

Within the female group (n=34), six (17.64%) had experienced COVID-19 gap of less than 10 days, 18 (52.94%) patients had gap of 10 to 20 days, whereas 10 (29.41%) had gap for more than 20 days. In the overall cohort of 105 individuals, 22 (20.95%) patients demonstrated a duration of less than 10 days, 62 (59.04%) patients exhibited a duration of 10 to 20 days, and 21 (20.00%) presented with a duration exceeding 20 days between COVID-19 and mucormycosis.

The categorization of patients as per oxygen requirement during hospitalization according to gender is presented in Table 2. Among the males (n=71), no oxygen support was required in 15 (21.12%) patients whereas 40 (56.33%) had required oxygen from an oxygen mask/nasal prong, 12 (16.90%) patients received bi-level positive airway pressure (BIPAP) therapy, and 5 (7.04%) were subjected to mechanical ventilation. In the female group (n=34), six (17.64%) patients had no oxygen requirement, 20 (58.82%) needed oxygen by mask/nasal prong, five (14.70%) underwent BIPAP therapy, and two (5.88%) patients required mechanical ventilation. In the comprehensive patient cohort of 105 individuals, 60 (57.14%) required oxygen by oxygen mask/nasal prong, 17 (16.19%) received BIPAP therapy, and seven (6.66%) underwent mechanical ventilation (Table 2).

Among the male patients (n=71), six (8.45%) reported exposure to damp areas, 13 (18.30%) had contact with soil or manure, five (7.04%) were engaged in animal husbandry activities, and rest five (7.04%) were involved in construction work. In the female group (n=34), two (5.88%) reported exposure to damp areas, six (17.64%) had contact with soil or manure, one (2.94%) was engaged in animal husbandry, and four (11.76%) were involved in construction work. Cumulatively, within the entire patient cohort of 105 individuals, exposure to damp areas was reported by eight (7.61%) patients, contact with soil or manure was experienced by 19 (18.09%) patients, animal husbandry activities were reported by six (5.71%) patients, and engagement in construction work was reported by nine (8.57%) individuals. Overall, exposure factors were recorded in 42 (40%) patients (Table 3).

**Table 3:** Gender wise distribution of environmental risk factors and organ involvement

		Male (71)	Female (34)	Total (n=105)
Exposure	Damp area	06 (8.45%)	02 (5.88%)	08 (7.61%)
	Contact with soil/manure	13 (18.30%)	06 (17.64%)	19 (18.09%)
	Animal husbandry	05 (7.04%)	01(2.94%)	06 (5.71%)
	Construction worker	05 (7.04%)	04 (11.76%)	09 (8.57%)
	Total	29 (40.84%)	13 (38.23%)	42 (40%)
Organ	Eye	41 (57.74%)	18 (52.94%)	59 (56.9%)
	Nasal Mucosa	26 (36.61%)	12 (35.29%)	38 (36.9%)
	Paranasal Sinus	46 (64.78%)	19 (55.88%)	65 (61.90%)
	Brain	15 (21.12%)	06 (17.64%)	21 (20%)
	Palate	08 (11.26%)	02 (5.88%)	10 (9.52%)
	Lungs	04 (5.63%)	01 (2.94%)	05 (4.76%)

Among the male group (n=71), paranasal sinus (64.78%), eye (57.74%), and nasal mucosa (36.61%) were frequently affected. In the female group (n=34), paranasal sinus (55.88%), eye (52.94%), and nasal mucosa (35.29%) were prevalent. Across the cohort (N=105), paranasal sinus (61.90%), eye (56.9%), and nasal mucosa (36.9%) showed significant involvement. Brain (20%), palate (9.52%), and lung (4.76%) were also involved with lesser frequency (Table 3).

In male category (n=71), common symptoms include headache in 38 (53.52%), orbital pain with restricted ocular movement in 32 (45.07%), proptosis in 31 (43.55%), and diminished vision in 31 (43.55%) cases. Among females (n=34), frequent symptoms were orbital pain with restricted ocular movement in 18 (52.94%), headache in 15 (44.11%), proptosis in 14 (41.17%), and diminished vision in 13 (38.23%) cases. Across the entire cohort (N=105), common symptoms were headache in 53 (50.47%), orbital pain with restricted ocular movement in 50 (47.67%), proptosis in 45 (42.85%), and diminished vision in 44 (41.90%). Other reported symptoms include fever, sinusitis, nasal discharge, skin redness, black eschar, jaw pain, eye redness, chest pain, cranial nerve palsy, and altered sensorium (Table 4).

**Table 4:** Genderwise distribution of presenting symptoms

Symptoms	Male (71)	Female (34)	Total (n=105)
Fever	11 (15.49%)	09 (26.47%)	20 (19.04%)
Nasal congestion	06 (8.45%)	04 (11.76%)	10 (9.52%)
Nasal discharge	16 (22.53%)	07 (20.58%)	23 (21.90%)
Headache	38 (53.52%)	15 (44.11%)	53 (50.47%)
Sinusitis	22 (30.98%)	10 (29.41%)	32 (30.47%)
Orbital pain with restricted ocular movement	32 (45.07%)	18 (52.94%)	50 (47.67%)
Redness of facial skin	11 (15.49%)	05 (14.70%)	16 (15.23%)
Black eschar	08 (11.26%)	04 (11.76%)	12 (11.42%)
Proptosis	31 (43.55%)	14 (41.17%)	45 (42.85%)
Diminished vision	31 (43.55%)	13 (38.23%)	44 (41.90%)
Cranial nerve palsy	02 (2.81%)	00 (0.00%)	02 (1.90%)
Jaw pain	08 (11.26%)	02 (5.88%)	10 (9.50%)
Redness of eye	05 (7.04%)	02 (5.88%)	07 (6.66%)
Chest pain	02 (2.81%)	01 (1.94%)	03 (2.85%)
Altered sensorium	01 (1.40%)	00 (0%)	01 (0.95%)

**Figure 1:**
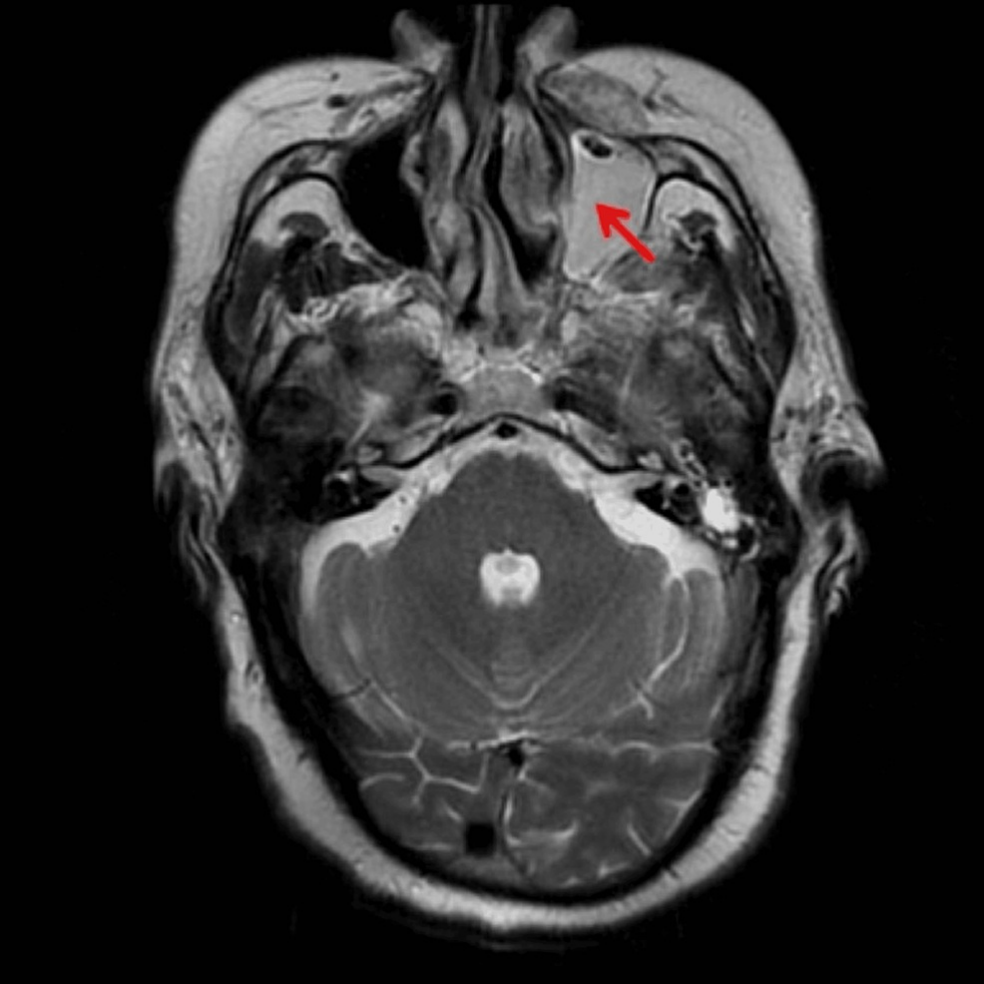
MRI (T1 image) reveals complete opacification of left maxillary sinus with homogenous high signal intensity (red arrow)

**Figure 2:**
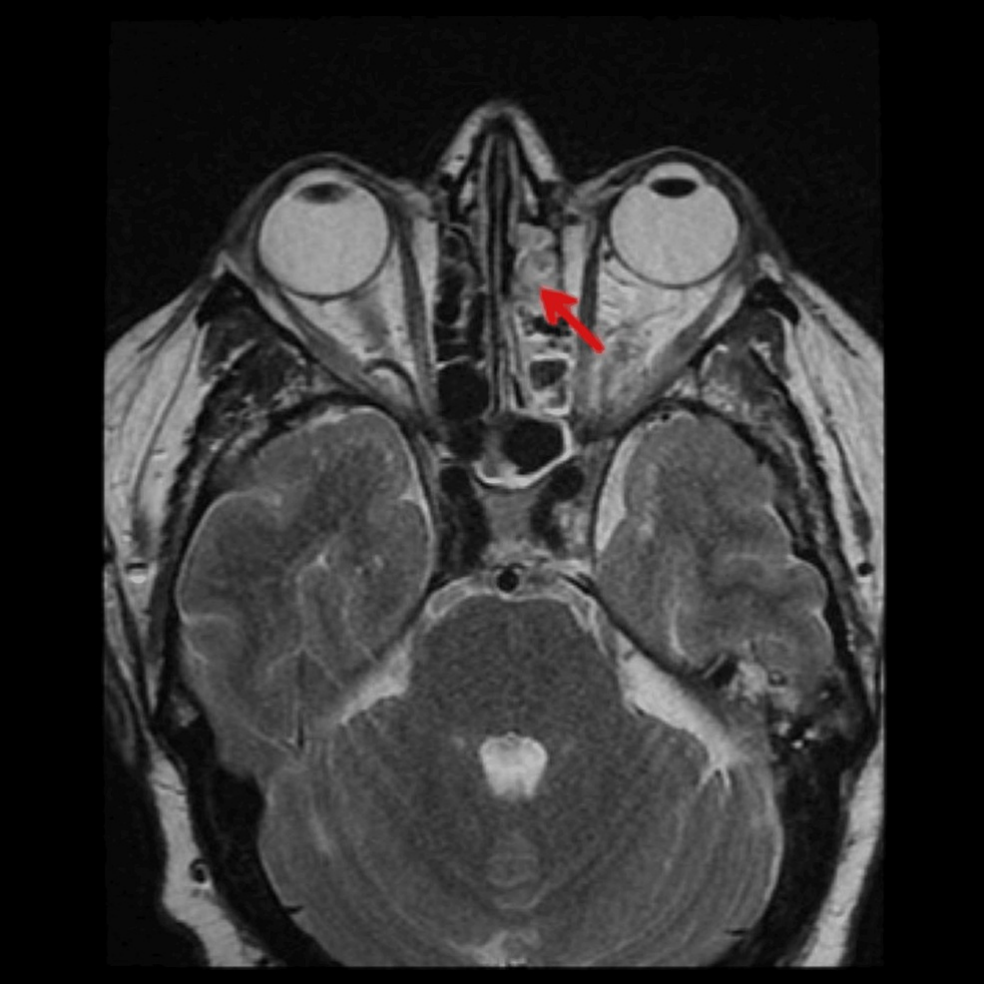
MRI (T1 image) showing mucosal thickening in the left maxillary sinus (red arrow)

**Figure 3:**
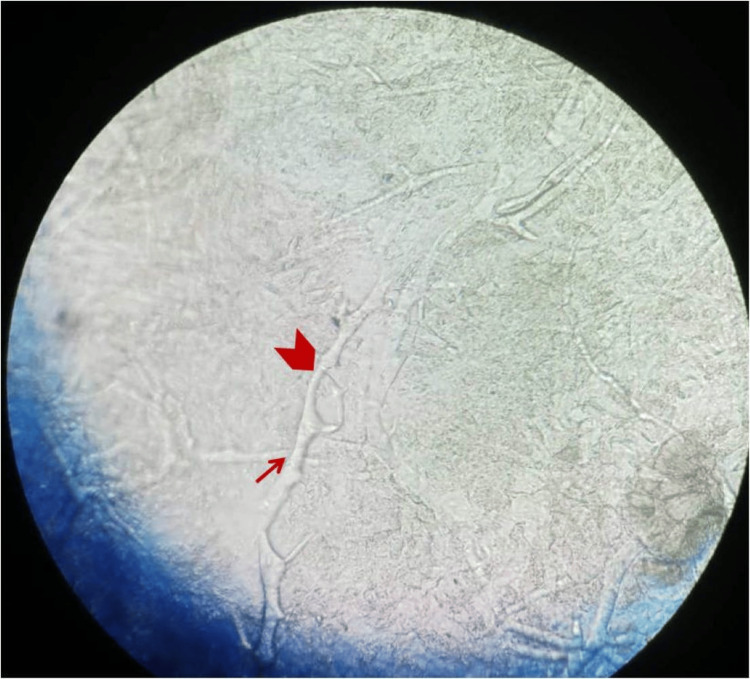
KOH mount-showed broad aseptate (arrowhead)/septate hyaline hyphae with wide-angle branching (arrow)

**Figure 4:**
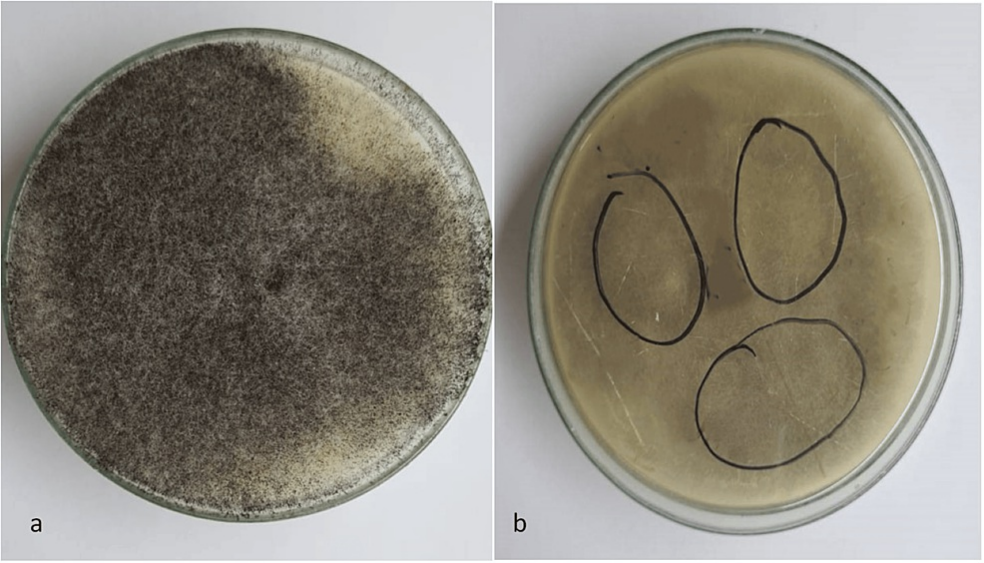
Rhizopus species (a) obverse side of culture plate-salt pepper-like growth and (b) reverse side of culture plate-pale shades of grey color colony

**Figure 5:**
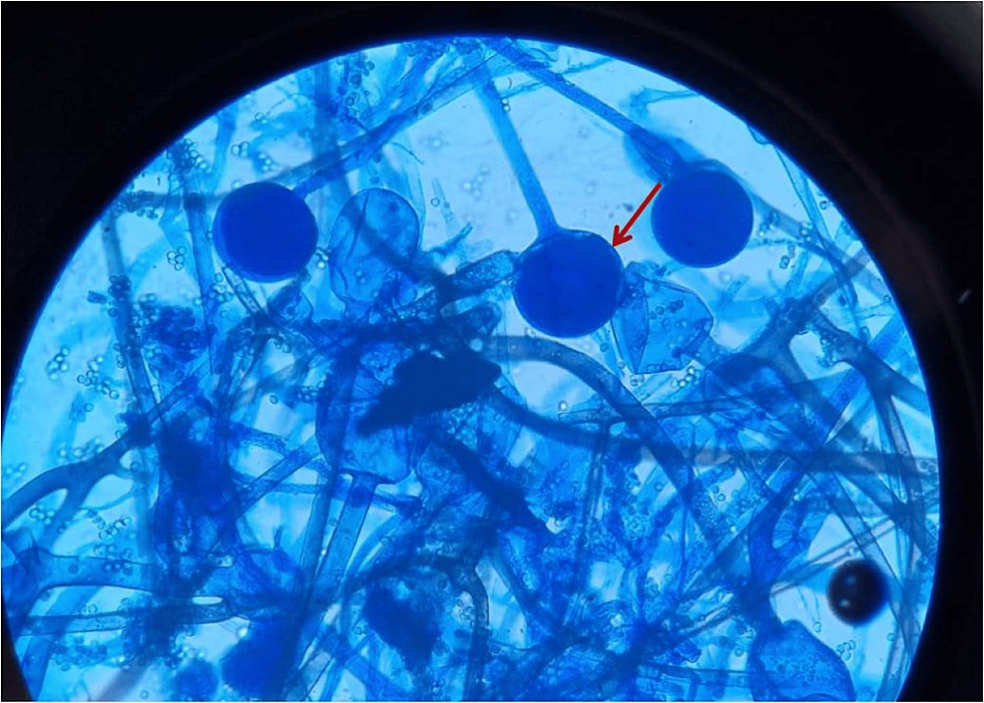
Lacto phenol cotton blue (LPCB) mount showed sporangium with sporangiophores (red arrow) and nodal rhizoids

Among the male patients, MRI/CT scans of the brain/paranasal sinus were undertaken in 62 cases and biopsies were performed in 28 cases. For confirmation of diagnosis of mucormycosis nasal wash was sent for every patient. Additionally, biopsies were performed in patients (n=28) who did not reveal the fungal hyphae or spores on the nasal wash sample. In the female group, MRI/CT scans of the brain/paranasal sinus were executed in 28 cases and biopsies were conducted in 12 cases. The endoscopy was done in all patients (Table 5). The extent of paranasal sinus, orbit, and intracranial involvement was assessed on an MRI/CT scan (Figures 1, 2). The tissue obtained from biopsy was sent for microbiological examination for identification of fungal species. The findings of the microbiological examination of Rhizopus species are depicted in Figures 3-5.

## Discussion

Mucormycosis is the third major cause of invasive fungal infection after Aspergillus and Candida species in humans. The prevalence of mucormycosis has been increasingly reported in patients with COVID-19 infection; however, not much data is presented for such patient’s clinicopathological features and outcomes [9].

The current study reports a male predominance for mucormycosis cases (67%) compared to females (33%). A similar finding was reported by Singla et al., who reported 68.80% of males and 31.30% of females with mucormycosis [10]. However, it is unclear why men were predominantly reported with mucormycosis. It has been proposed the protective role of estrogen in females is one of the preventive factors for a low prevalence rate [11]. The higher male prevalence was also observed in the study conducted by Anas et al., where mucormycosis affected 83.3% of males and 16.66% of females, respectively [12]. In addition, the age group between 41 and 50 years was most affected by mucormycosis (males; n = 22, and females; n = 13), and the mean age was 48.76 years. In a study done by Nair et al., the patients aged between 53.7 to 56.62 years were predominately diagnosed with mucormycosis [13].

Diabetes and irrational use of steroids were the most attributed risk factors for mucormycosis, accounting for 53.33% and 45.71% of the patients, respectively. Our study correlates with the findings of Patel et al. and Gupta et al., where uncontrolled diabetes (73.7%) and irrational use of steroids lead to prolonged ICU stay, and prolonged oxygenation therapy in more than 90% of the patients [14,15]. Singh et al. conducted a comparable study involving 101 mucormycosis patients and reported that 80% of the cases were associated with diabetes, and 76.3% had been administered corticosteroids [16].

In this study, all patients were literate which gave them an idea of the early understanding of disease and led to early visits to the health setup for further care. In the study done by Jayagayathri et al., 83% of participants knew about mucormycosis and 86% knew about it being an emergency [17]. Also, graduates were found to have better knowledge compared to the uneducated masses [17].

The duration between COVID-19 and mucormycosis was between 10 and 20 days in the majority of the patients 62 (59.04%), whereas 21 (20.0%) patients developed it after more than 20 days. Similar findings were reported by Kunhiparambath et al., with a mean average disease duration of 20.7±7.9 days from the onset of COVID-19 infection and mucormycosis [18]. The clinical course of the disease is dependent on factors such as comorbid conditions, early diagnosis, and treatment.

Various studies have reported administration of oxygen in 57%-90% of the patients with ROCM [19, 20]. In another study of patients with mucormycosis, 73% of the patients were administered oxygen [9]. Similarly, in our study, 57.14% of patients required oxygen therapy. The patients requiring mask/nasal prongs, NIV, and mechanical ventilators ranged from 59%-78%, 15%-19%, and 4%-7.1%, respectively [19,20]. Similarly in our study, 16.19% of the patients required non-invasive ventilators (NIV), 6.66% required mechanical ventilators, and the rest 77.15% required nasal prongs/face masks for oxygen administration. 

In our study, environmental risk factors such as damp area (7.61%), contact with soil/manure (18.09%), and animal husbandry (5.71%) were reported in patients. This can be attributed to ROCM being a fatal invasive fungal ailment affecting the nasal mucosa and paranasal sinuses. If timely intervention is not done, then it may infiltrate the orbit and brain due to angioinvasion and thrombosis. Rhizopus is the most common agent implicated in causing mucormycosis in India and is also associated with ROCM [21]. Rhizopus species are also found abundantly in soil and air samples [21].

Mucormycosis resulted in high involvement in the eye (56.9%), paranasal sinus (61.90%), and nasal mucosa (36.9%). In addition, the brain (20.0%), palate (9.52%), and lungs (4.76%) were reported subsequently. Cerebral involvement (20.18%) was reported by Singla et al. [10]. Similar findings of organ involvement were reported by Anas et al., with intra-orbital extension in 13 (43.3%) patients and intracranial extension in four (13.3%) patients [12]. Patients with mucormycosis were mostly diagnosed with symptoms such as sinusitis (30.47%), orbital pain with restricted ocular movement (47.67%), and proptosis (42.85%). Similar findings were reported by Sahu et al. for individuals demonstrating sinus and orbital involvement (n = 179). Prevalent symptoms include headache (n = 85, 47%) and facial and retro-orbital pain (n = 88, 49%). Notably, proptosis was the primary symptom among those with orbital involvement (n = 68, 39%), while 53(30%) patients experienced either blurred vision or substantial vision loss. For those with pulmonary involvement (n = 25), all cases presented with cough and dyspnea (100%), with 80% reporting chest pain and 35% experiencing hemoptysis [22].

This study reported the mortality of 34 patients (32.38%), with 23 males (22.0%) and 11 females (10.5%). This was similar to the finding of Singla et al., who reported an overall mortality rate of 33.9% [9]. Co-morbid conditions resulted in higher mortality, Amalanathan et al. reported 49% mortality rates among patients with diabetes and mucormycosis [23]. Sahu et al. also reported a mortality rate of 14% in patients presenting with diabetes or chronic kidney disease affected with mucormycosis [22].

Limitations

As some of the patients had earlier received home-based care during active COVID-19 infection recall bias in eliciting a history of preexisting diabetes and steroid use was present.

## Conclusions

ROCM was the most common presentation of mucormycosis between the second and third week of post COVID-19 infection. Diabetes mellitus and inadvertent use of steroids were major predisposing factors. Microbiological and radiological examination are important tools in assessing the etiology and burden of disease respectively. Therefore, a high degree of suspicion and early diagnosis with initiation of treatment is warranted in cases of mucormycosis in post COVID-19 infection.
